# Day 3 embryo transfer versus day 5 blastocyst transfers: A prospective randomized controlled trial

**DOI:** 10.4274/tjod.99076

**Published:** 2017-06-15

**Authors:** Şafak Hatırnaz, Mine Kanat Pektaş

**Affiliations:** 1 Private Bilge Hospital, Clinic of Obstetrics and Gynecology, İstanbul, Turkey; 2 Afyon Kocatepe University Faculty of Medicine, Department of Obstetrics and Gynecology, Afyon, Turkey

**Keywords:** Blastocyst, embryo transfer, in vitro fertilization, intracytoplasmic sperm injection

## Abstract

**Objective:**

This study aimed to show whether transferring day 5 embryos resulted in higher implantation and pregnancy rates than transferring day 3 embryos in Turkish women undergoing an intracytoplasmic sperm injection (ICSI) cycle.

**Materials and Methods:**

A total of 190 women who had ICSI after retrieval of more than four oocytes on the day of fertilization check were randomly assigned to undergo embryo transfer either on day 3 or day 5.

**Results:**

Day 3 and day 5 transfers were statistically similar with respect to the age of woman (p=0.107), duration of infertility (p=0.528), cause of infertility (p=0.850), number of collected oocytes (p=0.119), number of metaphase II oocytes (p=0.178), number of fertilized oocytes (p=0.092), and number of transferred embryos (p=0.556). The number of grade 1 embryos was significantly higher in day 5 transfers than in day 3 transfers (p=0.001). Day 3 and day 5 embryo transfers had statistically similar implantation, clinical pregnancy, twinning, and live birth rates (p=0.779, p=0.771, p=0.183, and p=0.649, respectively). The live birth rates in singleton pregnancies conceived after day 3 and day 5 embryo transfers were statistically similar (p=0.594).

**Conclusion:**

The efficacy of blastocyst transfer is not inferior to that of embryo transfer on cleavage stage. Performing blastocyst transfer may have benefits because it is associated with acceptable pregnancy rates and morphologic assessment on day 3 has limited predictive value for subsequent embryonic development.

## PRECIS:

Blastocyst transfer is an efficient embryo transfer method associated with satisfactory pregnancy outcomes, and performing blastocyst transfer may have benefits because morphologic assessment on day 3 has limited predictive value for subsequent embryonic development.

## INTRODUCTION

Intracytoplasmic sperm injection (ICSI) traditionally refers to the process of fertilization by combining an egg and sperm in the laboratory and then transferring the obtained embryos to the uterus. Embryo transfer (ET) performed on the second or third day of fertilization when the embryos are at the 4-8 cell stage has an implantation rate lower than 20%^(1,2,3)^.

In Turkey, ET is performed according to the regulations of the Ministry of Health. Single ET (SET) is recommended in women aged younger than 35 years who are having their first or second ET, whereas a maximum of two embryos can be transferred to women older than 35 years who have had 2 previous unsuccessful embryo transfers^(4)^. This restriction on ET reveals the need to increase the efficiency of *in vitro* fertilization (IVF) cycles. It has been hypothesized that blastocyst stage ET would permit the identification of embryos that could maintain their developmental process and, thus, allow the recruitment of good quality embryos with enhanced developmental ability. Therefore, it has been suggested that the blastocyst transfer at day 5 would succeed the highest pregnancy rates with the least number of embryos^(5,6,7)^. It is well known that growth and blastocysts at the 8-cell stage need a more complex environment, but the recent formulation of highly specialized media has permitted the preservation of blastocysts *in vitro*^(8,9,10)^.

The present study was arranged to determine whether transferring blastocyst-stage embryos would result in higher implantation and pregnancy rates than transferring cleavage-stage embryos in Turkish women undergoing ICSI cycles within the context of legal restrictions on the number of embryos being transferred. The findings of this study would correspond to the necessity of data regarding the implementation of blastocyst transfer in elective SET or double ET.

## MATERIALS AND METHODS

This prospective randomized study was conducted in accordance with the ethics principles outlined in the Declaration of Helsinki and approved by the local ethics committee (approval number: 149.01.2016).

### Patient selection and randomization

**Figure 1:**
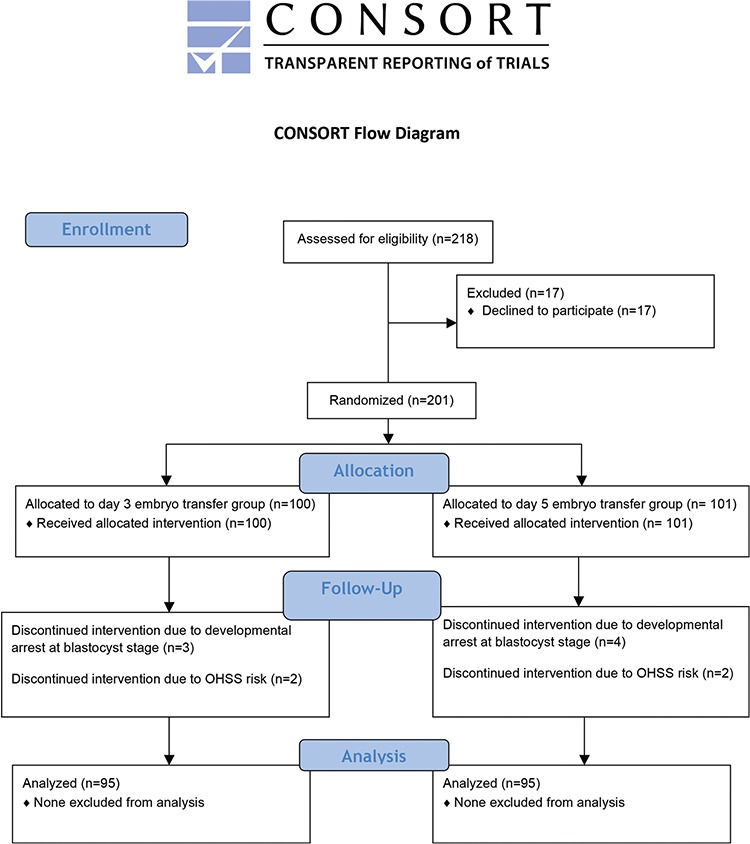
CONSORT flow diagram OHSS: Ovarian hyperstimulation syndrome

A total of 218 patients from whom at least four fertilized oocytes were retrieved on day 1 of fertilization (pronuclear scoring) during an ICSI cycle were eligible. The retrieval of at least four fertilized oocytes was set up as a criterion for eligibility to keep the risk for treatment discontinuation and cycle cancellation at a minimum. Women who were eligible were informed about the study. After the exclusion of 17 patients who refused to participate in the study, the remaining 201 patients gave written informed consent and were then randomized into either the day 3 embryo or day 5 transfer groups (n=100, n=101, respectively) using a computer-generated random number list. Once a patient was randomized, she remained in the same group throughout the study. Seven patients who discontinued their ICSI cycle due to developmental arrest at blastocyst stage and four patients who were thought high-risk for ovarian hyperstimulation syndrome were excluded from the study. Therefore, 95 patients in the day 3 ET group and 95 patients in the day 5 ET group were included in the final analysis (Figure 1).

### Controlled ovarian hyperstimulation and oocyte retrieval

The main stimulation protocol used was the long protocol. All patients receive leuprolide acetate (Lucrin^®^, Abbott Pharmaceuticals, IL, USA) on day 21 of the menstrual cycle. When menstrual bleeding began, transvaginal ultrasonography was performed and serum estradiol concentration was measured. As soon as ovarian quiescence was provided with the absence of ovarian cysts, and estradiol levels were below 50 pg/mL, the administration of gonadotropins was initiated with recombinant follicle-stimulating hormone at a daily dose of 150 IU (Gonal F^®^, Serono Laboratories, Randolph, MA, USA). The daily dose was adjusted by individual response. When at least three follicles reached a mean diameter of 17 mm, human chorionic gonadotropin [(HCG), Ovitrelle^®^, Merck-Serono, Aubonne, Switzerland] was administered. About 35-36 hours later, oocytes were retrieved using a double-lumen aspiration needle (Swemed Laboratories, Billdal, Sweden) under transvaginal ultrasonography guidance.

The follicular aspirate was poured into 60 mm Falcon dishes (Beckton Dickinson Labware, Franklin Lakes, NJ, USA) and cumulus-oocyte complexes were transferred into another dish with standard IVF medium (MediCult, Jillinge, Denmark). After the evaluation of each cumulus-oocyte complex for cumulus-corona cell morphology, the complexes were incubated in standard incubators until the time of removal of cumulus oophorus and corona radiata cells for ICSI.

### Sperm preparation

All semen samples liquefy for 15 to 30 minutes in an incubator. Sperm was prepared using a Percoll gradient (95 an 47.5%; Sigma, St Louis, MO, USA). One-half to 2 mL of raw semen was layered over the Percoll and the preparations were centrifuged at 300 g for 20 minutes. After centrifugation, the pellets from each tube were collected into 5 mL of culture medium and centrifuged at 1800 g for ICSI for 10 minutes. Fertilization was confirmed by the presence of two pronuclei and two polar bodies on day 1.

### Embryo grading

Embryos for day 3 transfer were cultured in the standard culture medium, whereas blastocysts for day 5 transfer were moved into G1.2 and G2.2 media (Scandinavian IVF Sciences, Gothenburg, Scandinavia) on day 1 and day 3, respectively. Standard culture medium was compared with sequential media because the use of G1.2 medium was not recommended for day 3 transfer. The number of blastomeres, the degree of fragmentation, and evenness of blastomere size for each embryo were recorded on days 2 and 3 for day 3 transfers and continued to be monitored on days 4 and 5 for day 5 transfers. A classification system introduced by Veeck was used for embryo assessment on the third day of culture. The embryos were classified as: grade 1, embryos with even blastomeres and no cytoplasmic fragments; grade 2, embryos with even blastomeres and minor cytoplasmic fragments or blebs; grade 3, embryos with uneven blastomeres and no or few cytoplasmic fragments^(11)^. Blastocysts were graded according to the size of blastocele cavity as early, full or expanded; the inner cell mass; and trophectoderm layer distribution.

### Embryo transfer

One or two top-grade embryos from both groups were transferred into the endometrial cavity on day 3 and 5. In case of developmental delay on day 5, one or two of the most advanced embryos were transferred into the uterus on day 5. Women aged ≥35 years and/or those who had two previous unsuccessful cycles had the right to two embryos being transferred. The luteal phase was supported by 50 mg intramuscular progesterone in oil once daily (Progestan^®^, Koçak Farma, İstanbul, Turkey) and estradiol, two 100 μg transdermal patches (Estraderm TTS^®^, Novartis Pharma AG, Basel, Sweden) with daily replacements. A pregnancy test was performed on the 12^th^ day following ET. Women with a positive HCG underwent a transabdominal ultrasound scan 15 days later. Clinical pregnancy was defined as the identification of an intrauterine embryo with heart beat.

### Statistical Analysis

The sample size was computed at the beginning of the study using an online analyzer (http://clincalc.com/Stats/SampleSize.aspx). For an expected difference of 15% between the two groups (10% vs. 25%) with a 5% level of significance and a power of 80%, a total of 200 patients (100 patients per group) were needed. Collected data were analyzed using the Statistical Package for Social Sciences version 18.0 (SPSS IBM Software, Armonk, NY, USA). Kolmogorov-Smirnov test, Fisher’s exact test, and the chi-square test were used for statistical analyses. Two-tailed p values <0.05 were regarded as statistically significant.

## RESULTS

Forty-nine women (25.8%) had no previous ICSI cycles, and 67 women (35.3%) had one previous ICSI cycle, and 74 women (38.9%) had at least two previous ICSI cycles.

Table 1 demonstrates the clinical characteristics of the day 3 and day 5 ETs. Day 3 and day 5 transfers were statistically similar with respect to the age of the women (p=0.107), duration of infertility (p=0.528), cause of infertility (p=0.850), number of collected oocytes (p=0.119), number of metaphase II oocytes (p=0.178), number of fertilized oocytes (p=0.092), and number of transferred embryos (p=0.556). When compared with day 3 transfers, the number of grade 1 embryos were significantly higher in day 5 transfers (p=0.001).

Table 2 displays the pregnancy outcomes of the day 3 and day 5 ETs. Day 3 and day 5 ETs had statistically similar implantation, clinical pregnancy, and live birth rates (p=0.779, p=0.771, and p=0.649, respectively). The miscarriage and perinatal death rates were also statistically similar in day 3 and day 5 ETs (p=0.551 and p=0.407, respectively). Day 3 and day 5 ETs resulted in statistically similar twinning rates (p=0.183). Additionally, the miscarriage, perinatal death, and live birth rates in singleton pregnancies conceived after day 3 and day 5 ETs were found statistically similar (p=0.276, p=0.083, and p=0.594, respectively).

SET and double ETs at day 3 had statistically similar pregnancy (39.0% vs. 58.3%; χ^2^=3.367; p=0.067) and live birth rates (32.2% vs. 44.4%; χ^2^=1.440; p=0.230). Single and double blastocysts transfers at day 5 also had statistically similar pregnancy (43.6% vs. 45.0%; χ^2^=0.017; p=0.895), and live birth rates (36.4% vs. 32.5%; χ^2^=0.152; p=0.696). SETs at day 3 and day 5 yielded statistically similar pregnancy (39.0% vs. 43.6%; χ^2^=0.254; p=0.614), and live birth rates (32.2% vs. 36.4%; χ^2^=0.219; p=0.640). Double ETs at day 3 and day 5 also resulted in statistically similar pregnancy (58.3% vs. 45.0%; χ^2^=1.348; p=0.246), and live birth rates (44.4% vs. 32.5%; χ^2^=1.146; p=0.284).

## DISCUSSION

SET has been instituted as a governmental policy in many European countries including Turkey. The reason for adopting such a policy is to avoid multiple pregnancies. Thus, the IVF centers dealing with SET have focused on identifying top quality embryos and designating the optimal time for its transfer. In other words, an embryo with the highest chance of implantation should be selected to achieve satisfactory pregnancy rates^(1,2,3,4)^.

It has been reported that morphologic assessment and grading of day 2 or 3 embryos have limited predictive value for further embryonic development^(12,13,14)^. Related studies have also indicated that recruitment at the blastocyst stage yields better results than selection at day 3, which merely depends on the morphologic evaluation of embryos. These studies also claim that pregnancy rates of up to 50% can be acquired by the transfer of blastocysts when compared with embryo transfer at the cleavage stage^(15,16,17,18,19,20,21)^. Similarly, it has been shown that the risk of aneuploidy was significantly lower in day 5 embryos than in day 3 embryos^(22)^. However, it has not been conclusively specified that blastocyst transfers have better perinatal outcomes than day 3 ETs^(23)^. In fact, a prospective observational study showed that about 50% of embryos conceived in 224 IVF/ICSI cycles were exposed to developmental arrest at the blastocyst stage^(24)^.

The rationale of blastocyst transfer is based on increasing the probability of obtaining advanced embryos with the highest chance for survival, i.e., implantation. The prolongation of embryo culture to day 5 requires a relatively high number of top quality blastocysts. Good quality cleavage-stage embryos increases the likelihood of good quality blastocyst embryos. Therefore, it would be prudent to expect no advantage if only a few good quality blastocysts exist in the culture^(20,25,26)^.

The transfer of two blastocysts at day 5 was more favorable than two embryos at day 3 in a cohort of 164 infertile women aged <37 years in a randomized controlled trial. In that study, transfers at blastocyst stage resulted in significantly higher pregnancy (51.3% vs. 27.4%) and live birth (47.5% vs. 27.4%) rates than the transfers at the cleavage stage. Yet, the twinning rate was statistically similar for the day 3 and day 5 transfers (36.8% vs. 30.4%)^(19)^. In accordance with this result, a prospective randomized study indicated that single blastocyst transfers had significantly higher pregnancy rate (23.7% vs. 35.6%) than the SETs in a cohort of 227 infertile women aged <36 years and who were on their first or second IVF trials^(26)^.

Contrary to the study above, another prospective randomized study reported that day 3 and day 5 transfers yielded statistically similar overall implantation (21% vs. 23%), pregnancy (39% vs. 39%) and twinning (11.9% vs. 15%) rates in a cohort of 201 infertile women^(27)^. Likewise, day 3 and day 5 transfers resulted with statistically similar implantation (47.4% vs. 45.3%), clinical pregnancy (46.3% vs. 44.2%), live birth (36.8% vs. 34.7%), and twinning (4.2% vs. 8.4%) rates in this study.

A review of related literature pointed out that day 5 ET with expanded blastocysts had a significantly higher implantation rate than non-expanding or non-cavitating embryos and, thus, single blastocyst transfer might be more successful than SET^(26,27)^. For instance, about 60% of women who received single blastocyst transfers had at least one excellent quality blastocyst and the pregnancy rate was 40.8% for these patients in a Belgian study^(26)^. Another study conducted in Saudi Arabia demonstrated that the success of day 5 transfers was a continuum of the number of good quality cleavage stage embryos and the availability of at least one blastocyst for transfer^(27)^. Correspondingly, the blastocyst transfers at day 5 could end up with higher implantation rates than early-stage ETs if at least one blastocyst is available for day 5 transfers.

The present study failed to detect statistically significant differences in implantation, clinical pregnancy, and live birth rates of day 3 and day 5 transfers. This inability can be attributed to the relatively small cohort size and relative heterogeneity in study population. The variations in clinical characteristics of the patients (i.e., age, cause of infertility, duration of infertility) might have caused such discrepancy.

### Study Limitations

Relatively small sample size for statistical analysis is the limitation of the study.

## CONCLUSION

In conclusion, the efficacy of blastocyst stage ET is not inferior to cleavage stage ET. Performing blastocyst transfer may have benefits because it is associated with satisfactory pregnancy rates and the assessment of embryo morphology at day 3 has limited predictive value for subsequent developmental process. The maintenance of embryo culture until day 5 may be a more sensible approach for the correct identification of best quality embryos with the highest probability of success for implantation.

The findings of the present study imply that blastocyst transfer at day 5 can be adopted as a reasonable approach in Turkish women who are to undergo elective SET or double ET. In order to maximize the probability of conceiving a pregnancy in an elective blastocyst transfer, this practice should be offered for ICSI cycles, in which several good quality embryos are obtained at day 3. However, patients who are to receive blastocyst transfer at day 5 should be informed that there is a greater risk of cycle cancellation because the embryo culture is extended to the blastocyst stage. Further research is warranted to determine the efficacy of blastocyst transfer at day 5 in Turkish women undergoing elective SET or double ET.

## Figures and Tables

**Table 1:**
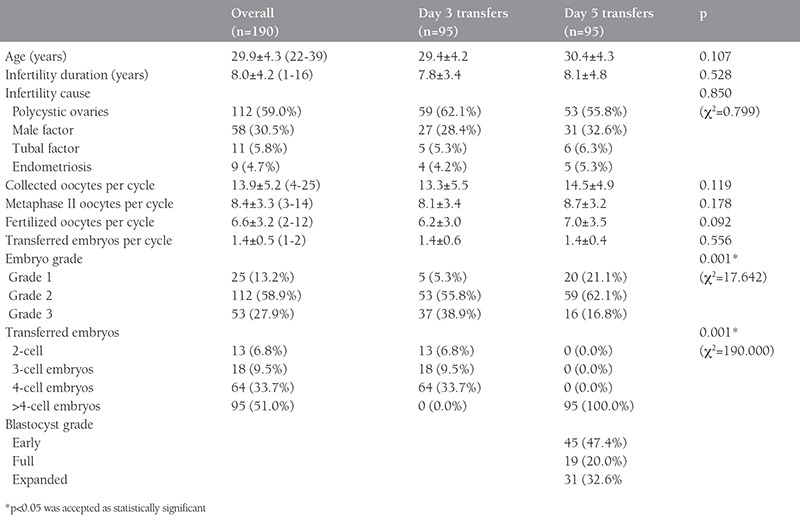
Clinical characteristics of the embryo transfers

**Table 2:**
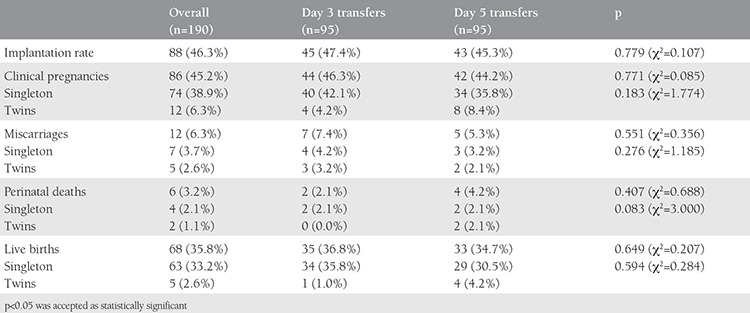
Pregnancy outcomes of the embryo transfers
